# Analysis of Internal Crack Healing Mechanism under Rolling Deformation

**DOI:** 10.1371/journal.pone.0101907

**Published:** 2014-07-08

**Authors:** Haitao Gao, Zhengrong Ai, Hailiang Yu, Hongyan Wu, Xianghua Liu

**Affiliations:** 1 State Key Laboratory of Rolling and Automation, Northeastern University, Shenyang, China; 2 School of Mechanical, Materials & Mechatronic Engineering, University of Wollongong, New South Wales, Australia; 3 School of Mechanical Engineering, Shenyang University, Shenyang, China; Washington State University, United States of America

## Abstract

A new experimental method, called the ‘hole filling method’, is proposed to simulate the healing of internal cracks in rolled workpieces. Based on the experimental results, the evolution in the microstructure, in terms of diffusion, nucleation and recrystallisation were used to analyze the crack healing mechanism. We also validated the phenomenon of segmented healing. Internal crack healing involves plastic deformation, heat transfer and an increase in the free energy introduced by the cracks. It is proposed that internal cracks heal better under high plastic deformation followed by slow cooling after rolling. Crack healing is controlled by diffusion of atoms from the matrix to the crack surface, and also by the nucleation and growth of ferrite grain on the crack surface. The diffusion mechanism is used to explain the source of material needed for crack healing. The recrystallisation mechanism is used to explain grain nucleation and growth, accompanied by atomic migration to the crack surface.

## Introduction

In recent years, steel production has increased rapidly, and it is significant to develop high performance steel products with low cost for high competiveness [1]. The initiation, propagation and possible healing of cracks in workpieces during the rolling process is not only related to the quality of the rolled pieces, but also affects the service life and usefulness of the rolled products. Undetected cracks in the product can bring about potential safety hazards when the product is put in use. Factors such as localized tensile stress and stress concentration result in crack initiation and propagation during rolling. On the other hand, conditions such as high temperature, compressive stress and diffusion are conducive to crack healing. The factors that affect crack propagation may result in micro cracks extending to macro cracks. On the contrary, macro cracks probably close gradually, and may heal completely under the right conditions.

Some researchers have studied the possibility of elimination of the internal defects in metals through heat treatment [2] and electropulsing [3]. Elimination of internal damage in metallic workpieces can lead to improved performance and even prolong the useful life of the product. Zhou et al [4] studied the healing influence of electropulsing on a steel workpiece with internal cracks. They found that localized cracks can be healed by electropulsing, while keeping the original micro-structure in regions without cracks unaffected. Zhang et al [5] provided evidence of diffusive healing of micro-cracks caused by inter-granular fatigue in iron during annealing. Wei et al [6], [7] carried out simulations of crack healing in Body-Centered Cubic (BCC) Fe and created a model of nano-scale crack evolution in iron. They also studied crack healing in 1045 steel using a method involving plate collision to heal preset cracks [8]. Yuan et al [9] studied crack healing in samples with preset cracks using a press machine. They concluded that the cavities caused could be filled and that the preset internal cracks may disappear under the condition of thermoplasticity. Yu et al [10]–[12] analyzed the behavior of cracks in a slab rolling process by Finite Element Modeling (FEM). Yu et al [13] also carried out experiments to investigate the internal crack healing under hot plastic deformation. However, an investigation of the process of grain recrystalisation around the crack zone has not been reported to date.

In this paper, a new experimental method, called the ‘hole filling method’, is proposed to simulate the healing of internal cracks inside rolled workpieces. The healing of internal cracks in a steel plate during rolling was studied. It is proposed that material diffusion, grain nucleation and recrystallization contribute to the healing of internal cracks. Based on the current research, a new concept of crack contact depth can be proposed to determine the critical condition for crack healing. Analytical expressions for the critical contact depth and the critical deformation rate for crack healing are proposed.

### Experimental Investigation

Plain carbon steel workpieces were used as the test samples. The chemical composition of the material is listed in Table 1.

**Table 1:** Chemical composition of rolled steel (mass fraction, %).

C	Si	Mn	S	P	Fe
0.20	0.25	0.90	0.01	0.02	remainder

**Figure 1:**
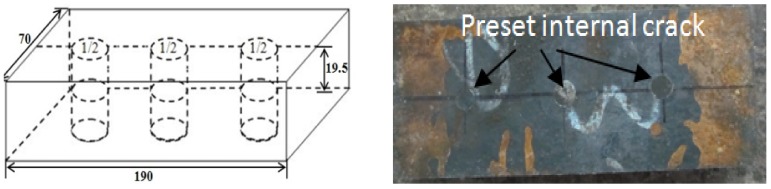
Illustration of preset internal crack in workpiece before rolling.

Figure 1 illustrates a workpiece with preset internal cracks before rolling. The small gap between the end faces of the two rods simulated the crack, as shown in Figure 1(a).

The specimen size of the test sample was 190 mm (length) ×70 mm (width) ×39 mm (thickness). Three internal ‘cracks’ halfway across the thickness were simulated in the rolled workpiece, using the ‘hole filling’ method. The diameter of the ‘cracks’ was 10 mm. Two short 10 mm diameter rods of the same material were knocked into each of the three holes lightly by way of interference fit. The small gap between the end faces of the two rods simulated the parallel cracks. The preset cracks were ground with #100 to #1200 grade abrasive papers and washed with alcohol. To prevent oxidation at the interfaces, and to prevent the rods from falling during heating and rolling, the narrow annular gaps on the sample surface were welded before hot rolling, shown in Fig. 1(b).

The rolling experiment was carried out using a hot rolling mill with 450 mm diameter rolls and a rolling speed of 1.5 m/s. The samples with preset cracks were heated to 1250°C, maintained at this temperature for one hour, and hot-rolled using one pass. The rolling temperature was 1100°C. After rolling, the samples were cooled slowly to 100°C, for about 16 hours. The cooling rate was therefore about 50°C/h to 60°C/h. The rolling experiment parameters are shown in Table 2.

**Table 2:** Experimental parameters.

Sample No.	Thickness before rolling(mm)	Thickness after rolling(mm)	Reduction (mm)	Reduction ratio(%)
1	39	31.2	7.8	20
2	39	23.4	15.6	40
3	39	19.5	19.5	50
4	39	15.6	23.4	60

## Results and Discussion

### Driving force behind internal crack healing

Compared with material without cracks, existing cracks result in an increment in the free surface energy in metals. The surface energy and the changes in the matrix microstructure introduced by the cracks are accompanied by lattice distortion, dislocations and empty seats. Based on the minimum energy principle, it will be relevant to seek a way to minimize the free energy during the process of crack healing. The free surface energy increment of the system introduced by cracks can be identified as the internal driving force behind crack healing.

Crack healing can be looked upon as a problem in thermodynamics. To accelerate crack healing, energy should be provided to overcome the resistance to crack healing, along with the required material. Some previous research suggests that the role of an external power supply is especially important [4], [14]. Surface tension at the crack surface and the greater atomic diffusion activation energy needed for eliminating lattice distortion are the main barriers to crack healing. When the sum of external driving force and the internal driving force is greater than the resistance to crack healing, the cracks could heal. Xiao et al [15] proposed a comparative model of internal crack healing in materials based on the assumption that the driving force is greater than the resistance to healing and crack instability extension theory in fracture mechanics.

**Figure 2:**
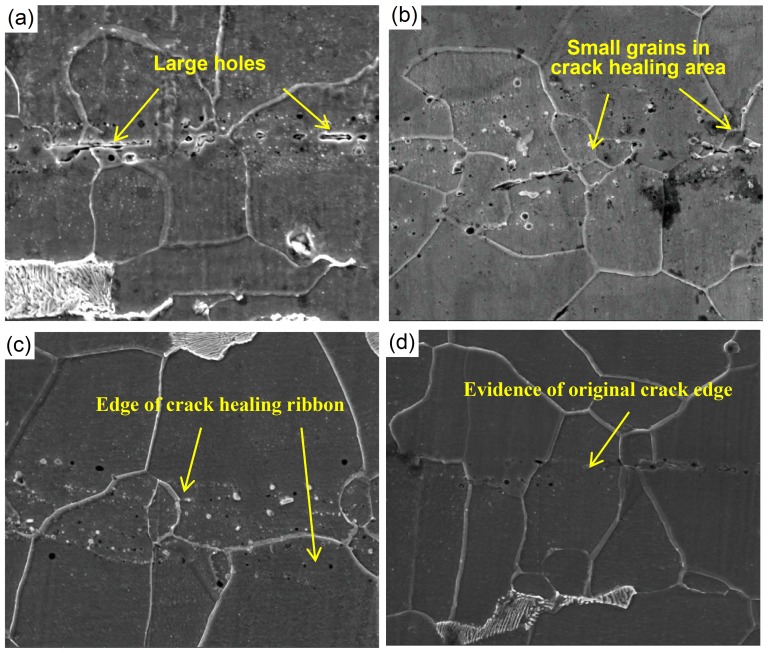
SEM image of crack healing under different reduction rates (a) 20%, (b) 40%, (c) 50%, and (d) 60%.

The external energy input in rolling comprises the energy of plastic deformation caused by rolling pressure and the heat transfer during rolling. As shown in Figure 2, the degree of crack healing increases gradually with an increase in the reduction rate. When the reduction rate is 20%, a distinct tendency for healing is seen in the crack region. Opposite sides of cracks are brought into almost complete contact, but some large voids are also seen in the crack region. With a reduction rate of 40%, the residual voids become smaller, and the grain size in the crack healing area is much smaller than elsewhere, shown in Figure 2(b). With a 50% reduction rate, voids are almost eliminated from the crack healing area. When the reduction rate reaches 60%, the microstructure in the crack healing area is seen to be uniform. The grain size in the crack healing area is larger than that corresponding to a smaller reduction rate and there is no noticeable distinction between the crack healing area and the original matrix. If the crack surfaces can join or be welded at high rolling temperatures, the atoms on opposite sides of the crack can interact. With increased deformation of the “convex” crack surface, greater contact between the crack surfaces is facilitated. The stress gradient and the lattice distortion will increase near the crack surface with an increase in the reduction rate, and thus the driving force of atom diffusion at the crack surface could increase.

**Figure 3:**
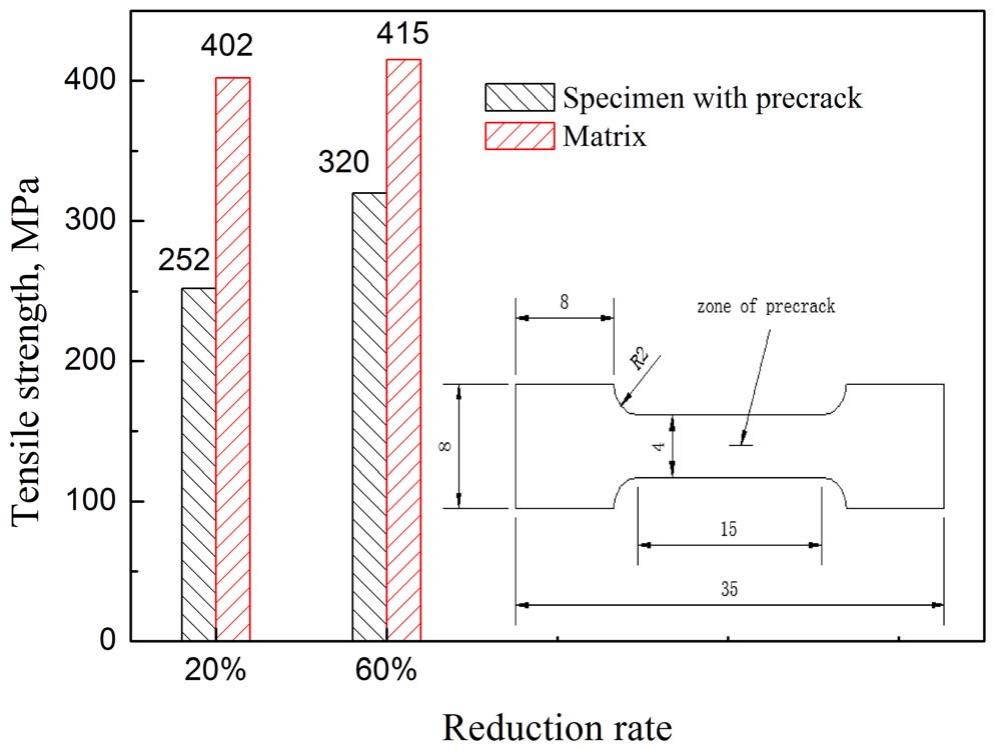
Tensile strength of matrix specimens and specimen with preset crack.

Tensile tests were carried out to verify that the extent of crack healing becomes greater with an increase in the reduction rate. Figure 3 shows the tensile stress for the specimens of the matrix (without crack) and specimens with precrack, corresponding to a reduction rate of 20% and 60% respectively. When the reduction rate is 20%, the tensile strength of specimens is 252 MPa for the preset crack samples, and 402 MPa for the matrix. When the reduction ratio increases to 60%, the tensile strength of specimens reaches 320 MPa for the preset crack samples while for the matrix it is 415 MPa. With the increase of strength in the crack healing area, the mechanical properties of the materials show an improvement.

### Grain nucleation and recrystallization behavior of internal crack healing

Meng et al [16] indicated that the reason for segmented internal crack healing was the non-uniformity of the degree of internal crack surface migrating to the crack interior. This was suggested by the macroscopic appearance of the uneven distribution of crack surface energy. Liu et al [17] confirmed the crack healing process sequence: (a) surface contact, (b) intermittent partial healing, (c) spread in the healing area and finally (d) complete healing. They proposed the concept of ‘crack contact depth’ as the critical condition of crack healing. An expression relating the critical contact depth and the critical deformation rate for crack healing were put forward, as shown in Equation (1):
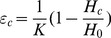
(1)


Here, *ε_c_* is the critical deformation rate, *H_c_* the critical contact depth, *H*_0_ the original contact depth and *K* the ‘amplification factor’.

**Figure 4:**
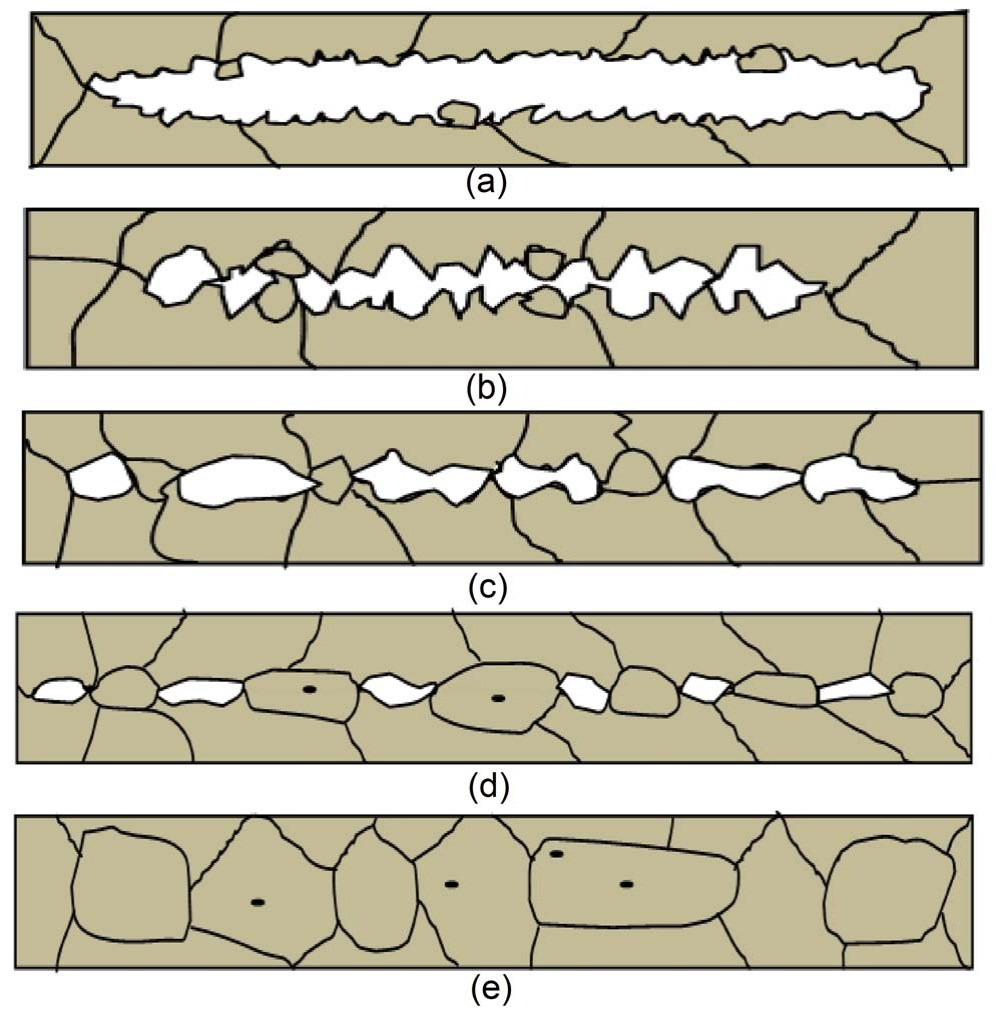
Crack healing process sequence, (a) recrystallization at the free crack surfaces, (b) shrinking the gap, (c) crack surfaces partially joined, (d) intermittent partial crack healing, (e) grains at the interface grow to matrix grains.

**Figure 5:**
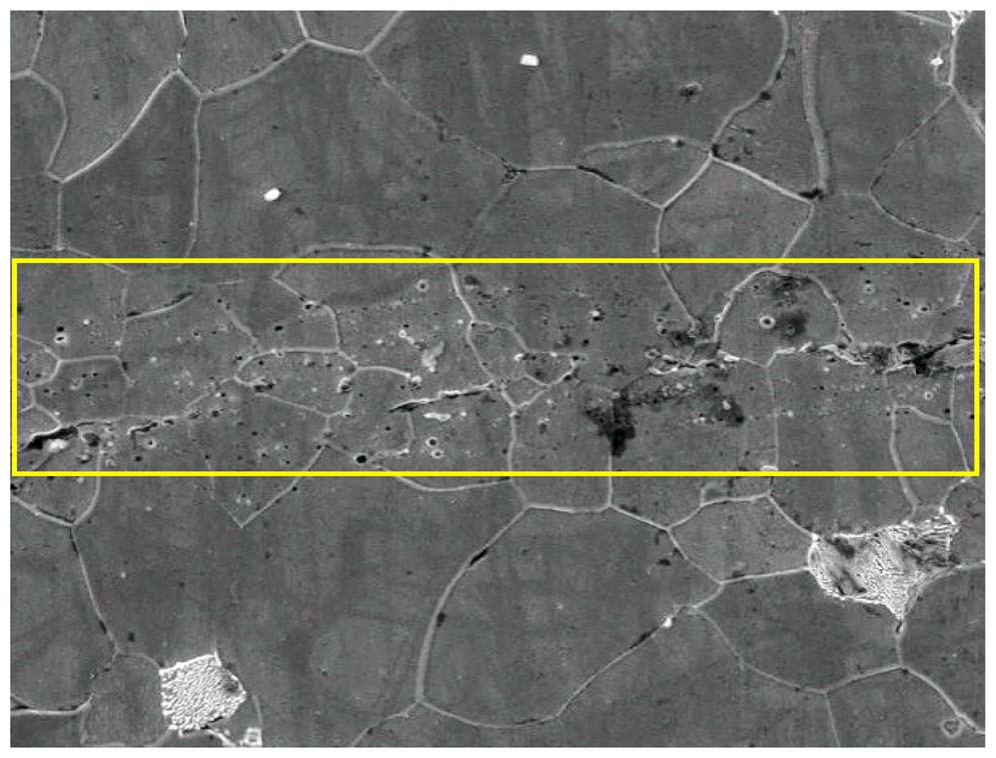
Distribution of porosity defects at crack healing zone.

The recrystallization in the ferrite and the grain growth during the process of crack healing are shown schematically in Figure 4. During hot rolling, high temperature combined with severe plastic deformation can induce crack healing. At high temperature, recrystallization can occur at the free crack surfaces of crack, as shown in Figure 4(a). With the energy input from severe plastic deformation, an uneven distribution of energy on the crack surface can lead to non-uniform deformation intensity on the crack surfaces. Tiny grains will appear and grow towards the gap, resulting in shrinking the gap gradually, shown in Figure 4(b). Usually, the surfaces of the crack are uneven and the convex surfaces come into contact first. In other words, crack healing is initiated at such points of contact. The crack surfaces can only be partially joined or welded to reach the threshold of atomic interactions under the rolling pressure, the remaining regions can be healed through atomic movement, as shown in Figure 4(c). Thus, the original long crack is divided into several shorter cracks. When the atoms on the opposite sides of the cracks come within the range of atomic interactions, intermittent partial crack healing will take place, and the crack healing area appears as a distribution of porosity defects, as shown in Figure 4(d) and Figure 5. The existence of a crack can increase the system energy, leaving the system in an unstable state. However, this excess energy can induce new grain nucleation and growth. New grains will form around these gaps. Tiny grains, appearing in local healing areas will continue to grow until their size approaches that of the matrix grains, shown in Figure 4(e).

**Figure 6:**
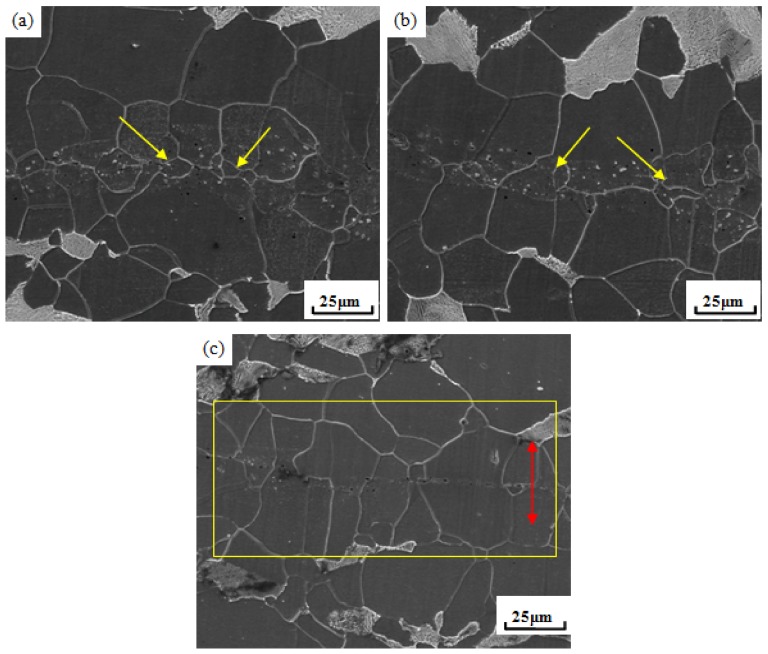
Recrystallization at the crack healing zone. (a) continuous recrystallization and merger, (b) recrystallization and merger with the matrix grain near the free surface, and (c) grains conduct directional growth to the pore until new grains grow up completely to realize crack healing.

As for the single channel rolling at high temperature as investigated in our research, the new grains carry on the recrystallization and merge with each other constantly (shown in Figure 6(a)) or with the matrix grain near free surface directly (shown in Figure 6(b)). The grains conduct directional growth to the pore until new grains grow up completely to realize crack healing, shown in Figure 6(c).

Based on the above analysis, the internal crack healing under rolling deformation appears to be controlled by both atomic diffusion and ferrite nucleation and growth on the internal crack surface. The atomic transfer from the matrix to the crack surface is caused by the former, which is the source of material supply in the crack healing area. The packing of atoms on the crack surface is caused by the latter, leading to the elimination of the crack. The effect of ferrite nucleation and growth on crack surface migration is reflected in two aspects. One results in segmented healing and the other accelerates crack healing. The ferrite nucleation and growth increase the difference in chemical potential between crack surface atoms and the matrix. During the atomic diffusion process with chemical potential gradient as the fundamental driving force, an increase in chemical potential will increase the diffusion flux and accelerate crack healing. The ferrite nucleation and growth on the crack surface facilitate the diffusion and accelerate the crack healing.

The diffusion mechanism can explain the source of the material needed to eliminate porosity under rolling deformation. The recrystallization mechanism can be used to understand the diffusion of material from the matrix to the defect-free surface and can be the reason for continued healing. The free surface will be activated with the temperature gradient and the free energy on defects free surface is greater than at the matrix. These provide energy for nucleation and recrystallization, leading to repair. At the same time the energy needed for material diffusion is provided by the temperature gradient.

### The role of atomic diffusion in internal crack healing

The atomic migration (diffusion) in a crystal can be of two types: (1) Chemical diffusion, caused by the uneven distribution of material in the crystal (concentration gradient), and (2) Self-diffusion, caused by thermal vibration. Atomic diffusion can also be caused by the prevalent stress field, thermal field and electric field. Zhang [18] put forward the view that diffusion is caused by different chemical potentials. Atomic diffusion occurs from high chemical potential to low chemical potential. The real cause of diffusion is not the concentration gradient, but the chemical potential gradient. The theory of the thermodynamics of diffusion is universal and can better illustrate the essence of the process. Only when atoms in solid solution at various points in the chemistry system are at the same potential, can the system reach thermodynamic equilibrium. Zhou [19] pointed out that internal crack healing was a process that involved material and energy exchange between an ‘open’ system and the surrounding environment. But he did not carry out the analysis in detail, only put it forward as a concept. In the current research, the specimens were heated in a furnace (endothermic process,), then taken out from the furnace for rolling (exothermic process). The specimens exchanged energy with outside environment (atmosphere outside the furnace) continuously. A number of studies indicate that the mechanism of internal crack healing in metallic materials mainly involves heat transfer and recrystallization. The heat transfer not only causes the micro defects to healed, but also results in nucleation and recrystallization of grains in the crack area. Atomic diffusion is crucial to the crack healing process, as it transports the necessary material to the crack healing area.

Not only is heat generated during the hot rolling process with plain carbon steel samples with internal cracks, the crack surfaces can come into contact or be ‘welded’ to reach the threshold of atomic interactions under the adequate rolling pressure. The large number of dislocations accumulated under plastic deformation can provide a rapid channel for atomic diffusion. During plastic deformation, a large number of atoms move from one equilibrium position to another across the crack. The atoms can come into contact with the atoms on the other side during the process of large numbers of atoms sliding or dislocations moving across the crack surfaces. If such contact is facilitated, the crystal lattice is formed and the crack is healed. The process of cooling after rolling can further facilitate the crack healing process.

Based on thermodynamics principles, the process of atomic interaction should tend to minimize the free enthalpy of the system. Two stages can be identified in the process of hot healing with internal cracks after rolling: (1) Movement of atoms from the matrix to the crack zone; (2) Joining of the crack surfaces. As the rolled workpieces are still at high temperature after rolling, a large number of vacancies will be formed during the process of slow cooling caused by the matrix atomic movement. When the concentration of vacancies is higher than the equilibrium vacancy concentration at that temperature, the atoms moving to the crack area can lead to an increase in the system enthalpy and the movement of atoms can continue. The atoms would be rearranged near the crack surface, resulting in the disappearance of internal crack surface, then ferrite nucleate on the free surface. The disappearance of distortion and surface energy near the surface caused by crack surface movement can bring about crack healing.

## Summary

The main findings in the study can be summarized as follows:

- Internal crack healing is driven by external and internal forces under rolling deformation. Plastic deformation and heat transfer are the external driving forces, while an increase in the free energy introduced by the cracks is the internal driving force for crack healing.
- Crack healing is controlled by diffusion of atoms from the matrix to the crack surface, and also by the nucleation and growth of ferrite grain on the crack surface. The material needed for internal crack healing is provided by the diffusion mechanism. The packing of atoms on the crack surface can be explained by the mechanism of recrystallization.

## References

[1] Liu XH, Zhi Y, Yu HL (2010) Rolling technology with reducing resources in China. Mater Manuf Process 25: 161–166.

[2] Li XG, Dong CF, Chen H (2002) Healing of hydrogen attack crack in austenite stainless steel under heat treatment. Acta Metall Sin (Eng ed) 15: 385–390.

[3] Zheng XG, Shi YN, Lu K (2013) Electro-healing cracks in nickel. Mater Sci Eng A 561: 52–59.

[4] Zhou YZ, Guo JD, Gao M, He G (2004) Crack healing in a steel by using electropulsing technique. Mater Lett 58: 1732–1736.

[5] Zhang HL, Sun J (2004) Diffusive healing of intergranular fatigue microcracks in iron during annealing. Mater Sci Eng A 382: 171–180.

[6] Wei DB, Han JT, Tieu KA, Jiang ZY (2004) Simulation of crack healing in BCC Fe. Scr Mater 51: 583–587.

[7] Wei DB, Jiang ZY, Han JT (2013) Modelling of the evolution of crack of nanoscale in iron. Comp Mater Sci 69: 270–277.

[8] Wei D, Han J, Jiang ZY, Lu C, Tieu AK (2006) A study on crack healing in 1045 steel. J Mater Process Technol 177: 233–237.

[9] Yuan CL, Zhong YX (2006) Self-healing mechanism of inner crack in plastic deformation under high temperature. J Plast Eng 13: 53–57.

[10] Yu HL, Liu XH, Li CS, Lan HF, Wang GD (2009) Research on the behavior of transversal crack in slab V–H rolling process by FEM. J Mater Process Technol 209: 2876–2886.

[11] Yu HL, Liu XH (2009) Thermal-mechanical finite element analysis of evolution of surface cracks during slab rolling. Mater Manuf Process 24: 570–578.

[12] Yu HL, Tieu K, Lu C, Deng GY, Liu XH (2013) Occurrence of surface defects on strips duirng hot rolling process by FEM. Int J Adv Manuf Technol 67: 1161–1170.

[13] Yu HL, Liu XH, Li XW, Godbole A (2013) crack healing in a carbon steel under plastic deformation. Metall Mater Trans A 45: 1001–1009.

[14] Zhao XP, Luo CR (1996) A model of intelligent material with self-repair function. Chinese J Mater Res 10: 101–104.

[15] Xiao YH, Wang JP (2008) Healing mechanism of the internal cracks in structural steel by heat treatment. Mater Mech Eng 9: 13–16.

[16] Meng FY, Nie SM (2010) Surface migration of internal crack in 45 steel during heating. J Plast Eng 17: 136–142.

[17] Liu XH, Yu HL (2009) Analysis of crack healing in hot rolled deformed piece. J Iron Steel Res 21: 36–39.

[18] Zhang YJ, Han JT, Liu JT, Yu WH (2008) Thermodynamic analysis on morphological evolution of inner crack healing process in metal materials. J Plast Eng 15: 122–125.

[19] Zhou BL (1997) Nonequilibrium processes in materlals processing. Chinese J Mater Res 11: 576–586.

